# Supplementary material for: The impact of hyperglycemia on prognosis in chronic obstructive pulmonary disease patients with coronary heart disease: A five-year prospective study
Source: PLoS One. 2025 Dec 5;20(12):e0335935. doi: 10.1371/journal.pone.0335935 (PMC12680245; doi:10.1371/journal.pone.0335935)
Supplement: Attachment — Submitted filename: Point-by-point response to reviewers..docx [file pone.0335935.s001.docx]

**The Impact of Hyperglycemia on Prognosis in Chronic Obstructive Pulmonary Disease Patients with Coronary Heart Disease: A Five-Year Prospective Study**

**Reviewer1**

We sincerely appreciate the opportunity to revise our manuscript and are deeply grateful for the reviewers' insightful comments, which have significantly helped improve the quality of our work. We have revised the article according to the comments of the reviewers and editors, and marked the modified parts in red font. The following are specific changes based on your suggestions.

SPECIFIC COMMENTS:

1. The rationale for specifically examining patients with both COPD and CHD, as opposed to those with either condition alone, needs to be more clearly articulated.

**We sincerely appreciate this invaluable comment. We apologize for the insufficient clarity in articulating the rationale for focusing on patients with both COPD and CHD. To address this, we have expanded the introduction and discussion sections to emphasize the unique clinical significance of this comorbid population. Epidemiological and pathophysiological rationale:** **COPD and CHD share common pathophysiological mechanisms and risk factors, including smoking, aging, and chronic systemic inflammation, leading to a high prevalence of comorbidity in elderly populations (20–30%). This coexistence is not merely additive but synergistic: acute COPD exacerbations may precipitate myocardial ischemia through hypoxemia, sympathetic activation, and increased ventricular afterload, whereas impaired cardiac output in CHD can exacerbate ventilatory-perfusion mismatch, worsening COPD-associated hypoxia and hypercapnia [Introduction, Lines 14-22]. Moreover, as you rightly pointed out, while the impact of hyperglycemia on the prognosis of patients with either COPD or CHD alone has been well-documented, its unique clinical implications in the context of comorbid COPD and CHD remain insufficiently explained. We greatly appreciate this valuable insight and have now incorporated a detailed analysis of this issue in the Discussion section [Lines 6-13] of our manuscript. Although the detrimental effects of hyperglycemia on clinical outcomes in patients with either COPD or CHD alone are well-established, its prognostic impact in the context of COPD-CHD comorbidity remains poorly characterized. Emerging evidence suggests that hyperglycemia may potentiate the pathophysiological interplay between these conditions, potentially through amplification of shared mechanisms including chronic low-grade inflammation, endothelial dysfunction, and metabolic dysregulation.**

2. A limitation noted is the absence of a comparative analysis with cohorts of patients having either COPD alone or CHD alone.

**We sincerely appreciate your insightful question, which was indeed a critical aspect we carefully considered during the initial design of our study. To begin with, our team collectively confirmed that the core focus of our research is to investigate the impact of hyperglycemia on the prognosis of patients with concurrent COPD and CHD. Through our preliminary literature review, we found that numerous studies have explored the effects of hyperglycemia in patients with either COPD alone or CHD alone, consistently concluding that hyperglycemia exerts significant adverse effects on disease progression and prognosis in these single-disease populations. Based on this well-established evidence, we acknowledged and defaulted to these prior findings, thus not including cohorts of patients with isolated COPD or isolated CHD to analyze the impact of blood glucose in the current study. However, research on the influence of hyperglycemia and its prognostic implications in patients with concurrent COPD and CHD remains largely unexplored and unreported in the existing literature. This identified gap underscores the innovative value of our chosen topic. We subsequently stratified patients with comorbid COPD and CHD into hyperglycemic and normoglycemic groups, and analyzed differences in baseline demographics, laboratory parameters, clinical characteristics, one-year acute exacerbation rates, and mortality between the two groups. This approach enabled us to derive clinically meaningful findings regarding the prognostic role of hyperglycemia in this comorbid population. Nevertheless, we fully agree with your valuable suggestion that incorporating cohorts of patients with isolated COPD or CHD would significantly enhance the comprehensiveness of our results, thereby better validating whether the observed prognostic effects arise from the synergistic interaction of the two coexisting diseases. We sincerely thank you for this constructive reminder. We have explicitly addressed this limitation in the discussion section of the manuscript [Discussion, The last paragraph, lines 12-19]. Furthermore, we aim to build upon your insights by designing prospective studies in the future to systematically compare the clinical characteristics and prognostic differences between patients with comorbid COPD+CHD and those with either disease alone.**

**Furthermore, you also suggested the need for a more in-depth exploration of how the presence of COPD uniquely modulates the interplay between hyperglycemia and CHD. We greatly appreciate this insightful recommendation. In response, we have conducted a renewed search of the latest literature in accordance with your feedback, delved deeper into the mechanisms underlying the unique modulation of the hyperglycemia-CHD relationship by COPD, and incorporated these findings into the discussion section of our manuscript** **[Discussion,** **Penultimate paragraph].** **COPD forms a unique pathophysiological network through the systemic effects of amplified systemic inflammation, hypoxia-oxidative stress crosstalk, and pulmonary vascular remodeling, thereby significantly potentiating the pathogenic role of hyperglycemia in CHD. Patients with COPD exhibit a persistent state of systemic inflammation, characterized by markedly elevated levels of proinflammatory cytokines such as IL-6 and TNF-α in the circulation. This chronic inflammation reinforces the pathogenic impact of hyperglycemia on CHD through two distinct pathways: metabolic derangement and accelerated vascular damage^29^. Studies have demonstrated that coronary artery plaques in COPD patients are more prone to calcification and instability, with hyperglycemia further increasing the risk of plaque rupture^30^. Furthermore, the characteristic pathological changes of COPD (e.g., emphysema, small airway remodeling) lead to chronic hypoxia, which, in conjunction with hyperglycemia, constitutes a dual hit to oxidative stress. Activation of hypoxia-inducible factors (HIFs) accelerates the proliferation of coronary artery smooth muscle cells and intimal thickening^31^. Additionally, research has revealed that pulmonary vascular remodeling results in pulmonary hypertension, compelling the right ventricle to increase its output. Such hemodynamic abnormalities severely impair coronary perfusion and augment cardiac workload^32^.**

3. While the inclusion of NHANES data is commendable, the relatively small sample size from the single-center Chinese cohort limits the generalizability of these specific findings.

**We sincerely appreciate this constructive reminder. The inclusion of NHANES data in our analysis was intended to compare with our local database, aiming to examine the prognostic impact of hyperglycemia in patients with concurrent COPD and CHD. Fortunately, our findings were consistent across both datasets: during the follow-up period, the incidence of acute exacerbations and mortality were significantly higher in the hyperglycemic group than in the normoglycemic group, with hyperglycemia identified as an independent risk factor for both outcomes in both our local cohort and the NHANES dataset. However, as aptly noted, the relatively small sample size of our single-center Chinese cohort limits the generalizability of these specific findings, which represents a key limitation of our study. Additionally, inherent differences in the baseline variables collected during the initial design of the two databases contributed to discrepancies in the prognostic factors identified between the analyses. Nevertheless, both datasets convergently confirmed that hyperglycemia is an independent risk factor for acute exacerbations and mortality. We greatly value your identification of these limitations, and we have incorporated these points into the discussion section of the manuscript as per your suggestions [Discussion,** **The sixth paragraph, lines 5-13]. In subsequent follow-up studies, we will continue to expand our database to minimize such discrepancies.**

All in all, we really appreciate your being so serious in reviewing the manuscript and giving so many precious suggestions. We have benefited a lot. We have made a lot of efforts to get this study published, and we hope to get your support. Thank you so much.

**Reviewer 2**

We sincerely appreciate the opportunity to revise our manuscript and are deeply grateful for the reviewers' insightful comments, which have significantly helped improve the quality of our work. We have revised the article according to the comments of the reviewers and editors, and marked the modified parts in red font. The following are specific changes based on your suggestions.

SPECIFIC COMMENTS:

1. In the normal glycemic group, it was stated that 13.86% of them had diabetes, in the other group were patients with > 7mmol/l glycemia, a value that diagnoses diabetes, but only 36.17% were diagnosed previously with diabetes. Please explain this disconcordance.

**We are particularly grateful for your identification of this valuable and meaningful issue. First and foremost, we assure you that all our clinical data are derived from patients' real - world information and medical records, ensuring their authenticity and reliability. However, when we analyzed this result, we were also filled with confusion and even hesitated whether to include it in the table, worrying that it might cause ambiguity. After analyzing this result as a team, we have the following explanations. In our study cohort, patients were divided into the normoglycemic group and the hyperglycemic group based on whether their FBG exceeded 7 mmol/L. The presence of diabetes in the normoglycemic group may be due to the fact that these patients had good blood glucose control, so their measured FBG did not exceed 7 mmol/L. Regarding the phenomenon that not all patients in the hyperglycemic group are diabetic, it should be considered that some patients may have stress - induced hyperglycemia, and hyperglycemia does not mean that all of them are diabetic patients. We sincerely appreciate your meticulousness and kindness in detecting and reminding us of this confusing issue. To avoid further ambiguity, we consider it more appropriate to delete the row of "Diabetes" in Table 1. We hope that our explanation and correction will meet your approval and satisfaction.**

2. There is no data about HbA1c or diabetes duration, diabetes treatment. Figure 2 is not fully supported by statistical data, please check again the interpretation.

**We greatly appreciate your excellent question. This limitation was briefly addressed in the discussion section of our manuscript, and we would like to elaborate further here. In our hospital, blood glucose levels are routinely measured via fasting capillary blood glucose testing. Typically, patients do not undergo a second measurement unless their fasting blood glucose exceeds 7 mmol/L, in which case a repeat test or HbA1c measurement is performed. Due to regulations set by the National Healthcare Security Administration, HbA1c is not a routine test and is only measured in patients with hyperglycemia or a confirmed diagnosis of diabetes. Regrettably, this prevented us from collecting HbA1c data for all patients to compare long-term glycemic control. However, in subsequent follow-up studies, we will expand our database and prospectively collect data on HbA1c, diabetes duration, and diabetes treatment regimens for further analysis. Additionally, you put forward an excellent suggestion to compare patient cohorts with and without diabetes. We are pleased to inform you that this analysis has been developed into a separate manuscript for submission, in which we found that COPD patients with comorbid diabetes have poorer prognoses. We hope that this subsequent study will have the opportunity to benefit from your guidance again and be successfully published. Furthermore, you kindly pointed out that the description of Figure 2 is insufficiently rigorous. We appreciate your meticulous reminder, as this indeed reflects an oversight on our part. We have revised the results section of the manuscript and re-described the content of Figure 2 accordingly. The Kaplan-Meier survival curves demonstrated that during our 12-month follow-up period, patients in the hyperglycemia group exhibited significantly higher rates of acute disease exacerbation compared to those in the normal blood glucose group (log-rank test, p=0.013; Figure 2A). Over the extended follow-up period exceeding 60 months, the hyperglycemia group showed markedly increased mortality relative to the normal blood glucose group (log-rank test, p=0.024; Figure 2B). Furthermore, our analysis of the NHANES database revealed that during an extended observation period of over 200 months, mortality rates remained significantly elevated in the hyperglycemic cohort compared to the normoglycemic controls (log-rank test, p=0.016; Figure 2C) [Results,** **The last paragraph, lines 28-39]. Please accept our sincere thanks once again.**

All in all, we really appreciate your being so serious in reviewing the manuscript and giving so many precious suggestions. We have benefited a lot. We have made a lot of efforts to get this study published, and we hope to get your support. Thank you so much.

**Reviewer 3**

We sincerely appreciate the opportunity to revise our manuscript and are deeply grateful for the reviewers' insightful comments, which have significantly helped improve the quality of our work. We have revised the article according to the comments of the reviewers and editors, and marked the modified parts in red font. The following are specific changes based on your suggestions.

SPECIFIC COMMENTS:

1. It is unclear whether the authors intended to evaluate the influence of stress-induced hyperglycemia or chronic exposure to hyperglycemia ( decompensated diabetes).

**Thank you sincerely for your insightful comments, which have helped us clarify critical methodological and interpretive issues in our manuscript. We appreciate your clarification on distinguishing stress-induced hyperglycemia from chronic hyperglycemia (decompensated diabetes). Our initial objective was to evaluate the prognostic impact of admission hyperglycemia (encompassing both stress-induced and chronic hyperglycemia) on patients with COPD and CHD, given that both phenotypes are common in acute hospital settings and linked to poor outcomes in comorbid populations. We acknowledge the limitation of using only admission fasting glucose to differentiate these subtypes. In our cohort, stress-induced hyperglycemia (e.g., due to acute exacerbations of COPD or cardiac events) likely contributed to the hyperglycemia observed in non-diabetic patients, while chronic hyperglycemia reflected underlying diabetes. However, due to the retrospective nature of our data, we lacked systematic measurements of stress hyperglycemia ratios (e.g., glucose/insulin ratio) or pre-admission glycemic profiles to rigorously distinguish these entities—this is a key limitation we have now explicitly noted in the discussion. Moving forward, we agree that incorporating stress hyperglycemia ratios and pre-admission HbA1c would enhance subtype classification, and we plan to include these metrics in future prospective studies.**

2. The definitions of the two groups for comparison included only an admission fasting glucose level of 7 mmol/L, and there is no clear explanation of how the patients were classified as having diabetes or not.

**We greatly appreciate your excellent question. This limitation was briefly addressed in the discussion section of our manuscript, and we would like to elaborate further here. In our hospital, blood glucose levels are routinely measured via fasting capillary blood glucose testing. Typically, patients do not undergo a second measurement unless their fasting blood glucose exceeds 7 mmol/L, in which case a repeat test or HbA1c measurement is performed. Due to regulations set by the National Healthcare Security Administration, HbA1c is not a routine test and is only measured in patients with hyperglycemia or a confirmed diagnosis of diabetes. Regrettably, this prevented us from collecting HbA1c data for all patients to compare long-term glycemic control. However, in subsequent follow-up studies, we will expand our database and prospectively collect data on HbA1c, diabetes duration, and diabetes treatment regimens for further analysis. Additionally, you put forward an excellent suggestion to compare patient cohorts with and without diabetes. We are pleased to inform you that this analysis has been developed into a separate manuscript for submission, in which we found that COPD patients with comorbid diabetes have poorer prognoses. We hope that this subsequent study will have the opportunity to benefit from your guidance again and be successfully published. Thank you sincerely once more.**

3. There is no data about HbA1c or diabetes duration, diabetes treatment. Figure 2 is not fully supported by statistical data, please check again the interpretation.

**We are particularly grateful for your identification of this valuable and meaningful issue. We sincerely appreciate your pointing out the methodological inconsistency between the lack of follow-up glycemic data and our discussion of chronic hyperglycemia. This discrepancy stems from our observational finding that admission hyperglycemia—regardless of its subtype—was associated with adverse outcomes, leading us to hypothesize that sustained hyperglycemia (either chronic or recurrent) may be the underlying mechanism. However, we agree that this inference remains speculative in the absence of longitudinal glycemic data. Additionally, we thank you for kindly noting that the discussion section was insufficiently comprehensive. In response, we have conducted a review of the latest literature and integrated it with our own research to re-examine, in depth, how COPD uniquely modulates the interplay between hyperglycemia and CHD through multidimensional mechanisms. These insights have been incorporated into the discussion section of the manuscript [Discussion, Penultimate paragraph]. “COPD forms a unique pathophysiological network through the systemic effects of amplified systemic inflammation, hypoxia-oxidative stress crosstalk, and pulmonary vascular remodeling, thereby significantly potentiating the pathogenic role of hyperglycemia in CHD. Patients with COPD exhibit a persistent state of systemic inflammation, characterized by markedly elevated levels of proinflammatory cytokines such as IL-6 and TNF-α in the circulation. This chronic inflammation reinforces the pathogenic impact of hyperglycemia on CHD through two distinct pathways: metabolic derangement and accelerated vascular damage^29^. Studies have demonstrated that coronary artery plaques in COPD patients are more prone to calcification and instability, with hyperglycemia further increasing the risk of plaque rupture^30^. Furthermore, the characteristic pathological changes of COPD (e.g., emphysema, small airway remodeling) lead to chronic hypoxia, which, in conjunction with hyperglycemia, constitutes a dual hit to oxidative stress. Activation of hypoxia-inducible factors (HIFs) accelerates the proliferation of coronary artery smooth muscle cells and intimal thickening^31^. Additionally, research has revealed that pulmonary vascular remodeling results in pulmonary hypertension, compelling the right ventricle to increase its output. Such hemodynamic abnormalities severely impair coronary perfusion and augment cardiac workload^32^.”We are particularly grateful for your valuable observation regarding the need to re-verify the proportion of patients with diabetes. First, we assure you that all our clinical data are derived from patients’ real-world information and medical records, ensuring their authenticity and reliability. When we initially analyzed these results, we were also puzzled and hesitated to include them in the table, concerned about potential ambiguities. After collective discussion, our team offers the following explanation: our cohort was stratified into normoglycemic and hyperglycemic groups based on whether fasting blood glucose (FBG) exceeded 7 mmol/L. The presence of diabetes in the normoglycemic group is likely attributable to good glycemic control, such that their measured FBG did not exceed 7 mmol/L. Regarding the finding that not all patients in the hyperglycemic group had diabetes, this may be explained by stress-induced hyperglycemia in some individuals, as hyperglycemia does not equate to diabetes in all cases. We truly appreciate your meticulousness and thoughtfulness in identifying and drawing attention to this confusing issue. To avoid further ambiguity, we deemed it more appropriate to remove the row labeled “Diabetes” from Table 1. We hope our explanation and revisions meet your approval. Your questions are exceptionally insightful, and we have gained tremendous knowledge from addressing them. We sincerely hope to have the opportunity to learn from you again in the future.**

All in all, we really appreciate your being so serious in reviewing the manuscript and giving so many precious suggestions. We have benefited a lot. We have made a lot of efforts to get this study published, and we hope to get your support. Thank you so much.
