# Supplementary material for: The impact of hyperglycemia on prognosis in chronic obstructive pulmonary disease patients with coronary heart disease: A five-year prospective study
Source: PLoS One. 2025 Dec 5;20(12):e0335935. doi: 10.1371/journal.pone.0335935 (PMC12680245; doi:10.1371/journal.pone.0335935)
Supplement: Attachment — Submitted filename: Point-by-point response to reviewers. 10.3.docx [file pone.0335935.s002.docx]

**The Impact of Hyperglycemia on Prognosis in Chronic Obstructive Pulmonary Disease Patients with Coronary Heart Disease: A Five-Year Prospective Study**

**Reviewer #3**

We sincerely appreciate the opportunity to revise our manuscript and are deeply grateful for the reviewers' insightful comments, which have significantly helped improve the quality of our work. We have revised the article according to the comments of the reviewers and editors, and marked the modified parts in red font. The following are specific changes based on your suggestions.

SPECIFIC COMMENTS:

1. Clarify the Methods - The abstract states that recruitment occurred in December 2016, with follow-up until March 2023. However, the Methods item Study population indicates that participant recruitment spanned from December 30, 2016, to March 5, 2023.

**Thank you very much for your kind reminder; this was an oversight on our part. As per your suggestion, we have revised the sentence in the Methods section from " The participant recruitment period spanned from 30/12/2016 to 05/03/2023" to align with the Abstract: " Recruitment for the study commenced in December 30, 2016, with follow-up until March 5, 2023. " Once again, we sincerely appreciate your careful attention in pointing out this error.**

2. All patients were followed up for a period exceeding 12 months. It would be beneficial to detail the methodology and to include data on the number of participants who were followed during such periods.

**Thank you very much for raising this question regarding the follow-up process; it has been truly helpful to us. We acknowledge that our Methods section did not provide sufficient detail on the implementation of follow-up at each time point, and the table also omitted the specific numbers of participants followed up. We have now supplemented both sections based on your kind reminder and suggestions. Specifically, we have added a detailed description of the follow-up procedures at each time point in the Methods section“We collected data on acute exacerbations of AECOPD during the first, third, and fifth years of follow-up, as well as all-cause mortality data throughout the entire follow-up period. The specific follow-up process was conducted by trained research assistants through telephone consultations or outpatient interviews with patients and their relatives (at least once per year). Data on annual exacerbations and survival status, along with outpatient and inpatient medical records provided by the patients, were uniformly recorded in a database, with data collected every three months.”. Additionally, we have included the exact number of participants followed up at each time point in Table 3. We greatly appreciate your help in making our research methodology more rigorous and complete.**

3. Table 3 presents the number of exacerbations and deaths in 1, 3, and 5 years. However, it lacks Kaplan-Meier curves for the study sample, as the subtitle suggests that Figure 2 pertains to the NHANES sample.

**Thank you very much for your kind reminder and suggestions. We suspect this is a beautiful misunderstanding. We would like to explain to you that Figure 2 is actually based on the results of both our study and the analysis of the NHANES database. Figures 2A and 2B are the Kaplan-Meier curves for the study sample, and Figure 2C is the Kaplan-Meier curve based on the NHANES sample. However, it might be that the previous title of Figure 2 was not clear and detailed enough, which led to your misunderstanding. We have now revised the title of Figure 2 to: "Figure 2. Kaplan-Meier curves for time to moderate-to-severe exacerbation (A), all-cause mortality (B), and NHANES-derived mortality (C) in patients with hyperglycemia and normal blood glucose groups during follow-up." Thank you again for pointing out this issue.**

4. In the discussion, the authors include some comments on the influence of some antidiabetic medications; however, despite the methods stating that the researchers collected the data using 'Self-administered questionnaires, clinical records, and self-reported data there seems to be no information on diabetes treatment that would be very important for discussion could be obtained.

**Thank you for your excellent suggestion, which very aptly pointed out the shortcomings in our work. As you rightly noted, the discussion section of our study does mention the impact of antidiabetic drugs, making data on diabetes treatment regimens crucial to that part of the discussion. Following your advice, we have supplemented Table 3 with information on the use of antidiabetic medications by patients in both the normal blood glucose group and the hyperglycemia group to make the research data more comprehensive. Additionally, we have correspondingly revised the description in the 'Results' section of the article [Results section, fourth paragraph, lines 1-5]. “Between the normal blood glucose group and the hyperglycemia group, the proportion of antidiabetic drug use was significantly higher in the hyperglycemia group (p < 0.001). No statistically significant differences were observed in the remaining symptom improvement indicators and treatment outcomes (Table 3).” We hope that our revisions now meet with your approval. Thank you once again!**

5. Avoid using definitive assertions such as "This study rigorously investigates the impact of hyperglycemia" and "Our research unequivocally demonstrates" as the methods do not allow such affirmation, considering the diagnosis of hyperglycemia in the hospital of the study uses routinely for glucose level evaluation a fasting capillary blood test.

**We sincerely thank you for providing such thoughtful and genuine feedback. The absolute statements used in our discussion were indeed inaccurate and inappropriate. We greatly appreciate you pointing out this shortcoming in a timely manner. We have revised the text according to your suggestions: “This study rigorously investigates the impact of hyperglycemia” has been changed to “This study preliminarily investigates the impact of hyperglycemia,” and “Our research unequivocally demonstrates” has been revised to “Our research demonstrates”. You also mentioned whether there is data to verify the diagnosis of hyperglycemia through blood tests. This limitation was briefly addressed in the discussion section of our manuscript, and we would like to elaborate further here. In our hospital, blood glucose levels are routinely measured via fasting capillary blood glucose testing. Typically, patients do not undergo a second measurement unless their fasting blood glucose exceeds 7 mmol/L, in which case a repeat test or HbA1c measurement is performed. Due to regulations set by the National Healthcare Security Administration, HbA1c is not a routine test and is only measured in patients with hyperglycemia or a confirmed diagnosis of diabetes. Regrettably, this prevented us from collecting HbA1c data for all patients to compare long-term glycemic control. However, in subsequent follow-up studies, we will expand our database and prospectively collect data on HbA1c for further analysis. Finally, you also suggested adding data related to blood glucose levels in the table of the results section. This was indeed an oversight on our part, and we have now included the median fasting blood glucose data for both the normoglycemic and hyperglycemic patient groups in Table 2. We have also incorporated these findings into the main text [Results section, Paragraph 3, Lines 11–13]: “Additionally, the FBG levels in the hyperglycemia group were significantly higher than those in the normal blood glucose group (p = 0.012)”. We really appreciate your being so serious in reviewing the manuscript and giving so many precious suggestions. We have benefited a lot.**

We are truly touched that, even during this second round of review, you have provided us with such valuable suggestions to help us continuously improve the quality of our paper. We are deeply grateful to you and sincerely hope to earn your approval this time. Thank you so much.
